# Assessing ‘no evidence of disease activity’ status in patients with relapsing–remitting multiple sclerosis: a long-term follow-up

**DOI:** 10.3389/fneur.2023.1187851

**Published:** 2023-08-07

**Authors:** Chiara Zilli, Pietro Scribani Rossi, Arianna Di Stadio, Mariangela Fratino, Giada Giuliani, Rosanna Annecca, Gaetano Russo, Vittorio Di Piero, Marta Altieri

**Affiliations:** ^1^Sapienza University of Rome, Rome, Italy; ^2^University of Catania, Catania, Italy

**Keywords:** NEDA, multiple sclerosis, DMT, relapse, EDSS, second-line treatment, MRI

## Abstract

**Introduction:**

Multiple Sclerosis (MS) is a chronic inflammatory demyelinating disease of the CNS with an autoimmune pathogenesis. Over the years, numerous disease-modifying therapies (DMTs) have proven effective in disease control; to date, there is a need to identify a personalized treatment effective in ensuring disease-free status or no evidence of disease activity (NEDA).

**Objective:**

identify clinical, demographic and treatment approach characteristics that affect the maintenance of NEDA-3 and the occurrence of clinical relapses during a 6-years follow-up.

**Materials and method:**

a retrospective study was conducted on a cohort of MS patients followed up with six-year period. All participants were treated with first- or second-line MS drugs.

Clinical relapse, NEDA-3 at 6 years and sustained EDSS were assessed as disease activity outcomes. Patients with follow-up of less than 6 years and insufficient clinical and radiological data were excluded from the study.

**Results:**

Two-hundred-eighty naive patients (mean age was 49.8 years, SD ± 11.35 years, 23–76, F/M 182/98), with MS were followed up for 6 years.

The mean age at diagnosis was 34.3 years (SD ±11.5, 14–62 years), the mean EDSS score at the onset was 1.9 (±1.3), 76.8% of patients had an EDSS below or equal to 2.5 at diagnosis.

In the cohort 37 (13.2%) directly received second-line treatment, 243 (86.8%) received first-line drugs.

The analysis showed that second-line treatment from beginning had a protective effect for the achievement of NEDA-3 (*p* = 0.029), on the prevention of clinical relapse (*p* = 0.018) and on number of relapses (*p* = 0.010); this finding was confirmed by logistic regression analysis (*p* = 0.04) and Kaplan–Meier analysis (*p* = 0.034).

**Conclusion:**

The results of this study demonstrate the efficacy of targeted and early intervention so as to act in the right time window, ensuring a favorable outcome in both clinical and radiological terms; this could be decisive in reducing clinical relapse, disease progression and related disability. Therefore, prescribing highly effective drug in the early stages of the disease represents a leading strategy with the most favorable cost–benefit ratio.

## Introduction

Multiple sclerosis (MS) is a chronic autoimmune disease of the central nervous system (CNS) characterized by inflammation, demyelination and degeneration of oligodendrocytes and their axons, mediated by the activation of cells of the immune system (1).

MS is the leading cause of progressive neurological disability and the first cause of neurological symptoms in young adults. Because of the high prevalence of MS in young population, some emerging disease-modifying therapies (DMTs) aiming to control symptoms have been developed; these new drugs have sophisticated capacities for modulating immune function (2).

Currently, the primary aim of treatment in MS is to reach “no evident disease activity” (NEDA) which is a composite of three related measures of disease activity: (i) no relapses; (ii) no disability progression and (iii) no MRI activity (such as new or enlarging T2 lesions or Gd-enhancing lesions) (3, 4).

DMTs are chosen by evaluating a few factors:

i) the control of neuro-inflammation to prevent accumulation of disabilities and prolong wellness;
ii) avoiding cognitive deterioration, fatigue and depression which are caused by MS and at the same time worsening the disease;
iii) the high heterogeneity of prognosis among patients;
iv) treatment adherence, which is strictly related to patients’ wishes and expectations (5).

Several elements must be considered about use of DMTs. Some are objectively standardized factors such as clinical and radiological criteria, and potential treatment efficacy, while others are more related to subjective interpretation such as cognition status, tolerability and/or poor adherence to treatment, comorbidities that may influence medication management and metabolism, personal factors that hinder the suspension or continuation of therapy, such as pregnancy. By means of the new drugs with different pharmacokinetic and pharmacodynamic profiles, it is today possible to choose a tailor-made treatment defining a patient’ s precision medicine.

The European Medicines Agency and the Italian Regulatory Agency has classified DMTs as first- or second-line, according to the risk/benefit profile found in clinical trials (6). (1) First-line therapy includes Interferons, Glatiramer acetate, Teriflunomide and Dimethyl-fumarate, (2) Second-line therapy includes Fingolimod, Ozanimod, Ponesimod, Siponimod, Natalizumab, Ocrelizumab, Ofatumumab, Cladribine and Alemtuzumab.

Second-line therapies are indicated for (1) patients with RRMS who have not responded to a complete and adequate course of therapy (6–12 months of treatment) with at least one DMT; (2) patients who from onset show a more aggressive form of the disease and/or unfavorable prognostic indicators, which justifies the use of stronger drugs with a lower safety profile.

To this aim in the present study, we evaluated the prevalence of patients who maintained NEDA-3 over a 6-year follow-up in relation to treatment and the variables linked to the recurrence of clinical relapses.

## Materials and methods

We retrospectively studied all patients attending the MS Centre of Sapienza University of Rome between January 1, 2016, through December 31, 2022. Data was collected from medical records and outpatient visits.

- The inclusion criteria for the study were:
- naïve patients with recent (within 6 months) diagnosis of MS according to the revised McDonald criteria (7);
- first or second-line treatment for MS started within 6 months of disease onset;
- regular clinical and radiological follow-up visits over time (at least 2 outpatients visit each year and 1 MRI 1.5 T annually);
- absence of comorbidity (overlap with other inflammatory diseases, neoplasms).

Exclusion criteria were as follows: diagnosis of MS > 6 months, being already under DMTs, shorter follow-up than 6 years, having a chronic disease that could potentially interfere with the length of follow up.

Relapses and EDSS scores were evaluated at 6-month intervals during the time of clinic visits. All patients underwent brain and spinal MRI (1.5 T) yearly.

Relapses were defined as either new signs or symptoms lasting more than 24 h without concurrent fever or illness, recorded by the attending physician during the 6-month face-to-face visit.

Disease progression was defined as an increase in EDSS score of 1 or more at the clinic visit and maintained at the subsequent visit. If the EDSS score was 0 at baseline, progression was defined as a sustained EDSS score change of 1.5 or more.

The outcome index used was the NEDA 3. In case of patients switching to a secondary progression (SP) course during the observation, they were assumed as NEDA failure due to EDSS progression (8–10).

During the observation period of our study some DMTs were not yet available such as: Ozanimod, Ponesimod and Ofatumumab. For this reason, they were not included in the analysis.

Treatment discontinuation was defined as a treatment-free period of 90 days or more (10). Alemtuzumab was an exception since a standard regimen consists of 2 short courses 1 year apart and no further treatment if patients remain clinically and radiologically stable (2).

### Magnetic resonance imaging

Patients performed MRI studies at one-year intervals. Standardized MRI (1.5 T) included at least T1-weighted axial and coronal imaging with and without Gadolinium enhancement, diffusion- weighted imaging (DWI), T2-weighted axial sequences as well as axial, coronary and sagittal T2 fluid-attenuated-inversion-recovery (FLAIR) imaging (11).

## Statistical analysis

Characteristics of the study population were presented using descriptive statistics. Mean and standard deviation (SD) values were calculated for continuous variables, whilst frequencies were reported for categorical variable. Age at onset of MS and EDSS score were also dichotomized as follows: age > 25 years and EDSS >2.5.

A multivariable logistic regression model was employed to assess association between demographic and clinical characteristics and NEDA at 6 years. Factors with *p* value <0.1 at univariate level were considered. The Odd Ratio (OR) and the relative 95% confidence interval (CI95%) were calculated. Kaplan Meier’s curves over 6 years were calculated in the two groups (induction or not) in relation to the lesion load, the sustained EDSS and the time to first relapse.

The software Stata was 16.0 was used for all analyses, a value of <0.05 was considered statistically significant.

## Results

During the time-interval 513 patients were evaluated. Among them, 233 patients were excluded from the analysis: 21 changed MS Centre, 37 never started DMT or received it >6 months from the disease onset, 66 discontinued DMT during the follow-up, and 110 had a follow-up <6 years.

The remaining 280 patients were then analyzed. The mean age was 49.8 years (SD ± 11.35 years, 23–76 years), F/M 182/98, mean age at diagnosis of 34.3 years (SD ±11.5, 14–62 years).

The mean EDSS score at the onset was 1.9 (±1.3), with a minimum of 0 and a maximum of 5,

76.8% (215) of patients had an EDSS below or equal to 2.5 at diagnosis; differences between EDSS at baseline and after six years as well as medication changes over time are shown in the Table 1.

**Table 1:** Clinical and demographic features at onset and at the end of follow-up.

Patients = 280	(*n*, % at onset)	(*n*, % at 6 yrs)	*p* value
Male	98, 35%		
Female	182, 65%		
Mean age/SD	49.8, 11.35		
Mean age at diagnosis/SD	34.3, 11.5		
Age > 25	202, 72%		
Mean EDSS (interval)	1.9, 0–5	2.83, 0–8.5	<0.001
EDSS >2.5	66, 23.6%	107, 38.2%	<0.001
**First-line DMT**	243, 86.8%	138, 49.3%	
Interferons	178, 63.6%	34.12%	<0.001
Teriflunomide	22, 7.9%	23, 8.2%	
Glatiramer acetate	27, 9.6%	38, 13.6%	<0.001
Dimethyl-fumarate	16, 5.7%	43, 15.4%	<0.001
**Second-line DMT**	37, 13.2%	147, 52.5%	
Fingolimod	10, 3.6%	79, 28.2%	0.003
Natalizumab	13, 4.6%	29, 10.4%	<0.001
Ocrelizumab	7, 2.5%	25, 8.9%	<0.001
Cladribine	6, 2.2%	14.5%	<0.001
Alemtuzumab	1, 0.3%	0	

During follow-up, 110 (39.3%) patients had at least one relapse and 38 (13.5%) had more than three relapses; on average this occurs after 37.3 months (±19.6), with a higher frequency (26%) in the first 36 months (Table 2) of all DMT patients, at the end of follow-up 144 (51.4%) were still on first-line therapy while 136 (48.6%) were on second-line therapy; in the latter group, 99 (72.8%) performed a vertical shift. Of the total patient sample, 37 (13.2%) directly received second-line treatment from baseline. Specifically, 99 (35.4%) patients underwent a vertical therapeutic shift, 78 (28%) due to both clinical and radiological recurrence, while 21 (7.5%) only due to the addition of new lesions to the RM. Over 6 years, 46.1% of patients changed more than one DMT (Table 2). The most represented clinical course was relapsing remitting (RR) in 210 (75%), SP in 51 (18.2%), and primary progressive (PP) in 19 (6.8%).

**Table 2:** Change of treatment during the 6-years follow-up.

DRUG	ONSET (*N*, %)		Last (*n*, %)		No shift (*n*, %)		Horizontal shift (*n*, %)		Vertical shift (*n*, %)	
**First-line DMT**	**243**	***86.8%***	**144**	***51.4%***	**69**	***69%***	**75**	***92.6%***	**99**	***100%***
Interferon	178	*63.6%*	35	*12.5%*	34	*34%*	63	*77.8%*	81	*81.8%*
Teriflunomide	22	*7.9%*	45	*16.1%*	13	*13%*	2	*2.5%*	7	*7.1%*
Glatiramer acetate	27	*9.6%*	22	*7.8%*	10	*10%*	9	*11.1%*	8	*8.1%*
Dimethyl-fumarate	16	*5.7%*	42	*15.0%*	12	*12%*	1	*1.2%*	3	*3%*
**Second-line DMT**	**37**	***13.2%***	**136**	***48.6%***	**31**	***31%***	**6**	***7.4%***	*/*	*/*
Fingolimod	10	*3.6%*	62	*22.1%*	8	*8%*	2	*2.5%*	*/*	*/*
Natalizumab	13	*4.6%*	29	*10.4%*	9	*9%*	4	*4.9%*	*/*	*/*
Ocrelizumab	7	*2.5%*	26	*9.3%*	7	*7%*	0	*0.0%*	*/*	*/*
Cladribine	6	*2.2%*	13	*4.6%*	6	*6%*	0	*0.0%*	*/*	*/*
Alemtuzumab	1	*0.3%*	3	*1.1%*	1	*1%*	0	*0.0%*	*/*	*/*
Siponimod	0	*0.0%*	3	*1.1%*	0	*N/A*	0	*N/A*	*/*	*/*

First-line and second-line treatment are highlighted in bold as these two groups are the focus of our study and in italic there are the percentage of this values.

After 6 years, NEDA-3 was reached by 106 (37.9%) patients. After 6 years, 54% (20/37) of naïve patients treated with second-line treatment from beginning achieved the NEDA 3 compared with only 35% (86/243) of those who started treatment with first-line drugs (*p* = 0.029). At the univariate analysis none of the clinical and demographic characteristics were statistically related to the achievement of NEDA 3 at 6 years; 61.4% of the patients have a current EDSS ≤2.5, and only 35% of the subjects had a sustained increase of at least one point in the EDSS compared to the previous 6 months: this supports the efficacy of DMTs in slowing disability progression.

**Figure 1:**
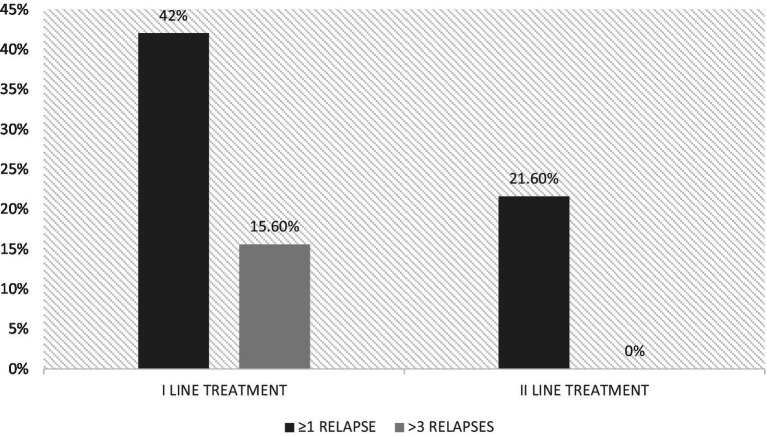
Percentage of relapses at the end of follow-up.

Only 8 (21.6%) naïve patients receiving second-line treatment from the beginning had relapses during the 6-year-follow-up, compared to 102 (42%) treated with first-line treatment (*p* = 0.018); among them, 38 had more than 3 relapses during the follow-up and none in the group that received a second-line treatment from the beginning (*p* = 0.01) (Figure 1).

At logistic regression analysis, none of the clinical and demographic factors reached statistical significance, except for initiation of treatment with a second-line drug (*p* = 0.04), which was protective (Table 3).

**Table 3:** Logistic regression analysis.

Factors	*B*	S.E.	*p* value	Exp (B)
Age > 25 years	−0.039	0.278	0.89	0.962
Sex (F)	−0.243	0.265	0.36	0.784
EDSS >2.5	0.053	0.298	0.86	1.05
Second-line DMT at onset	−0.812	0.395	0.040	0.444

**Figure 2:**
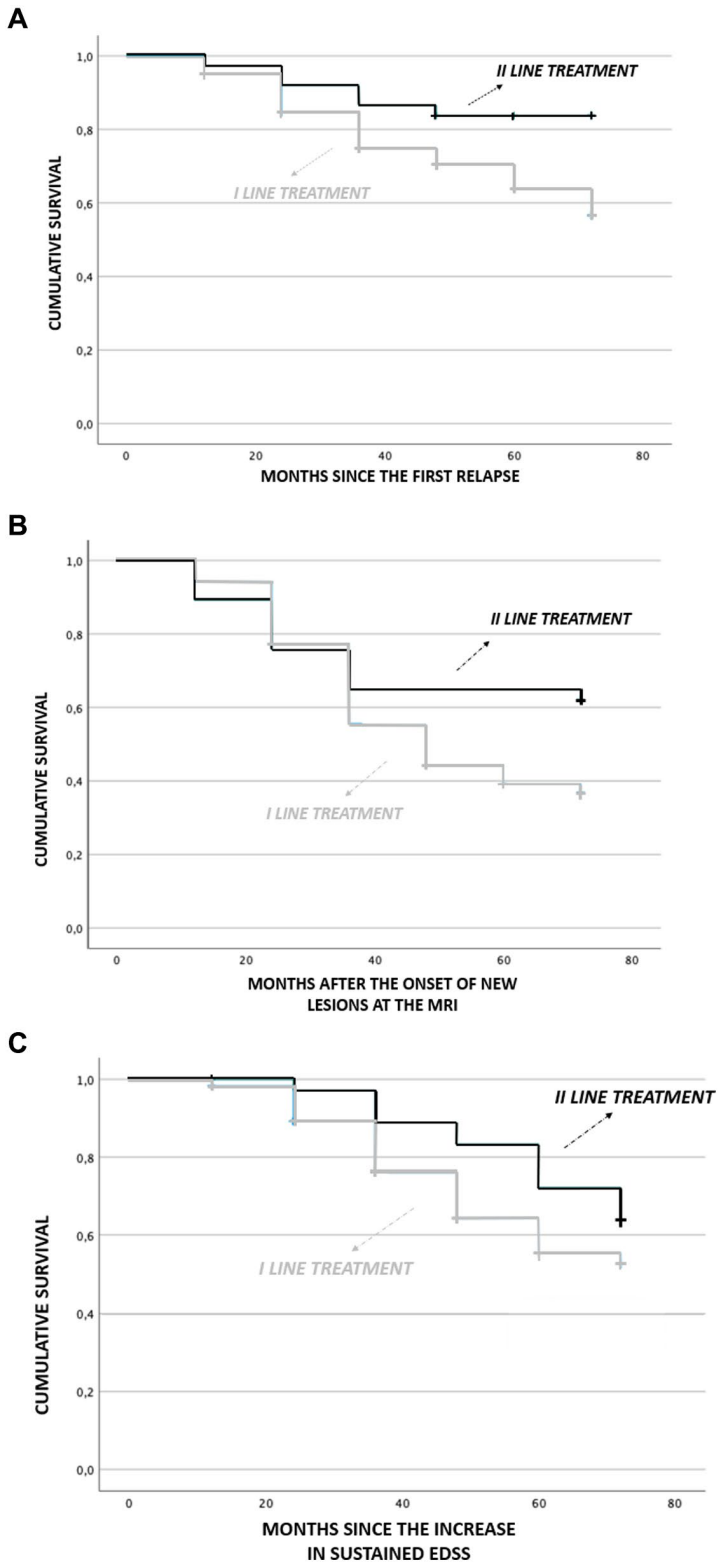
Comparison of survival analysis in patient treated with first-line treatment and second-line treatment. **(A)** months since first relapse, **(B)** months aaer onset of new lesions at MRI, and **(C)** months since increase in sustained EDSS.

Survival analysis showed earlier relapses in patients initially treated with first-line drugs than in those treated with second-line ones. This finding is even more evident in the long-term follow-up (*p* = 0.034). In particular, in the first months of the disease, the time to recurrence presentation is mostly similar between the two groups. After the first 12 months, a lower cumulative frequency of relapses in patients treated with second-line drugs becomes clear. After 36 months this difference is even more evident, while patients treated with second-line drugs reach a plateau, clinical recurrences continue to occur in patients treated with first-line drugs (Figure 2).

An earlier increase of MRI lesions (new T2 weighted and/or Gadolinium enhanced) was observed in patients initially treated with first-line drugs than in those treated with second-line ones (*p* = 0.023) while it was not significant for the sustained EDSS.

Concerning the radiological data in 146 patients was observed an increase of T2 weighted lesions during the follow-up. Among them, 62 (22%) were Gadolinium-enhanced.

## Discussion

In our study about 40% of patients achieved NEDA-3 at 6 years. This is in agreement with the literature. In a longitudinal cohort study of 219 patients with clinically isolated syndrome (CIS) or RRMS with mean disease duration of 6.6 years at study onset NEDA status declined over time with NEDA-3 present among 46.0% at 1 year (12). No demographic or MS variable including DMT at study baseline predicted NEDA-3 at 7 years. In a cohort of relapsing MS patients treated with natalizumab NEDA-3 was reached by 34% of patients at 7 years (13).

Apart from the choice of therapeutic approach, in our naïve patients with MS the only protective factor to relapses in the long-term was starting with a second-line treatment. Even though several subjects in first-line therapy were subsequently treated with highly effective second-line DMTs, differences in terms of outcome were still evident, suggesting that the use of the latter is more effective if started early. This result agrees with the current literature. Recent observational studies showed that early initiation of second-line treatment in RR-MS may provide more benefit that an escalation approach in both decreasing the risk of developing secondary progression and disability accrual, at least in terms of a 5–6 years of follow-up (14–16).

This is even more evident when one considers that patients who receive second-line drugs from the beginning are generally those with worst prognostic factors (2, 17).

A recent Italian study in a large naïve RR-MS population comparing the long-term effect of early versus late start of high-efficacy DMTs on disability found that those treated with second-line treatment experienced a slower disability progression compared to those who switched to them after a suboptimal response to moderately effective DMTs (15). This approach outdates the traditional “treat to target” method in which a moderately effective drug is first used, and more aggressive treatment is indicated with a breakthrough disease (evident clinically or by MRI) occurs.

It is commonly thought that starting with a first-line therapy represents a low-risk strategy, but the clinical application of this approach may be inadequate to prevent unfavorable long-term outcomes. Furthermore, the safety of this scheme may be called into question: escalation can be characterized by the sequential use of different DMTs with heterogeneous immunomodulatory or immunosuppressive actions which can lead to complex and variable effects on the immune system. The long-term consequences of this effect can be much more unpredictable than a single action.

The cost benefits analysis may lend a hand to a more aggressive approach in reducing drug switching and adverse effects (18). In Italy, where the use of DMTs is conditioned by economic and pharmacovigilance reasons, high-efficacy DMTs can be used as first-line therapy only if the patient is very active or considered at highest risk of accumulating disability. This approach would seem less advantageous from an economic point of view than one might think. In the long run, reducing the patient’s disability is more profitable also in economic terms. As a result, it is vital to identify situations for which physicians take the opportunity of escalating treatment when indicated (e.g., progression of disease determined by clinical relapses, EDSS disability score and imaging data) (19).

Making decisions in medical care is a complex task involving a variety of cognitive processes: decisions based on erroneous assessments may result in incorrect patient expectations, and potentially suboptimal advice, treatment, and prognosis. Moreover, many decisions are made with limited information from observational studies or clinical trials that may not apply to particular patients (20, 21). For example, patients at low risk of developing MS progression may prefer to avoid ‘risky’ treatments, whereas high-risk patients would prefer the most effective treatment even if need to take higher risks.

This study has some limits: its observational and retrospective nature may have introduced a number of detection and reporting bias. We attempted to mitigate these shortcomings by ensuring that all patients enrolled had standardized clinical and radiological follow up over time and those who did not meet these criteria were excluded from the study. Moreover, the follow-up protocols used in our study were largely comparable, and then, the magnitude of such a bias would be minimal. Our study provides real-world evidence, representative of clinical practice in a tertiary MS center with valuables insights into challenge of the therapeutic management in active MS.

Another limitation of the study is the radiology: MRI 1.5 T was used instead of a 3.0 T, and the lesion site data were not detailed. Only the addition of new lesions and the presence of gadolinium-enhanced lesions were reported in the registry, but not the site. In addition, NEDA 4 data on brain volume status were not provided.

Although NEDA-3 accounts for many aspects of disease activity, it may not adequately capture the neurodegenerative processes of MS, especially in early disease stages. Since brain atrophy is correlated with and predictive of longer-term disability progression and cognitive decline, we are aware that with NEDA-3 we have not evaluated all potential adverse factors on patient outcome.

## Conclusion

The results of this study suggest that prescribing high efficacy drugs early in the history of the disease represents a prominent strategy with the most favorable cost–benefit ratio. This is even more clear if we consider that clinical relapses are only the tip of the iceberg in terms of MS disease activity (16).

Increasingly numerous studies demonstrate the existence of a limited window of opportunity for an effective intervention. Further studies of this nature could shortly lead to an update of treatment guidelines and the elimination of the restrictions on starting with second-line drug, especially in the early stages of the disease, reducing both relapses and disability accrual in patients with more moderate MS.

Since the primary impact of DMT is observed in relapsing–remitting MS (RRMS), a separate study focusing specifically on this subset of patients would be highly valuable.

## Data availability statement

The original contributions presented in the study are included in the article/supplementary material, further inquiries can be directed to the corresponding author.

## Ethics statement

Ethical review and approval was not required for the study on human participants because clinical data was used. The patients/participants provided their written informed consent to participate in this study and use clinical data.

## Author contributions

MA and CZ wrote the article and collected the data. PS and GR entered the data in a database. GG and RA performed the statistical analysis. VP, AS, and MF revised the text. All authors contributed to the article and approved the submitted version.

## Conflict of interest

The authors declare that the research was conducted in the absence of any commercial or financial relationships that could be construed as a potential conflict of interest.

## Publisher’s note

All claims expressed in this article are solely those of the authors and do not necessarily represent those of their affiliated organizations, or those of the publisher, the editors and the reviewers. Any product that may be evaluated in this article, or claim that may be made by its manufacturer, is not guaranteed or endorsed by the publisher.
